# Regulators and Effectors of Arf GTPases in Neutrophils

**DOI:** 10.1155/2015/235170

**Published:** 2015-11-02

**Authors:** Jouda Gamara, François Chouinard, Lynn Davis, Fawzi Aoudjit, Sylvain G. Bourgoin

**Affiliations:** ^1^Division of Infectious Diseases and Immunology, CHU de Quebec Research Center, Quebec, QC, Canada G1V 4G2; ^2^Faculty of Medicine, Laval University, Quebec, QC, Canada G1V 0A6

## Abstract

Polymorphonuclear neutrophils (PMNs) are key innate immune cells that represent the first line of defence against infection. They are the first leukocytes to migrate from the blood to injured or infected sites. This process involves molecular mechanisms that coordinate cell polarization, delivery of receptors, and activation of integrins at the leading edge of migrating PMNs. These phagocytes actively engulf microorganisms or form neutrophil extracellular traps (NETs) to trap and kill pathogens with bactericidal compounds. Association of the NADPH oxidase complex at the phagosomal membrane for production of reactive oxygen species (ROS) and delivery of proteolytic enzymes into the phagosome initiate pathogen killing and removal. G protein-dependent signalling pathways tightly control PMN functions. In this review, we will focus on the small monomeric GTPases of the Arf family and their guanine exchange factors (GEFs) and GTPase activating proteins (GAPs) as components of signalling cascades regulating PMN responses. GEFs and GAPs are multidomain proteins that control cellular events in time and space through interaction with other proteins and lipids inside the cells. The number of Arf GAPs identified in PMNs is expanding, and dissecting their functions will provide important insights into the role of these proteins in PMN physiology.

## 1. Introduction

**Figure 1:**
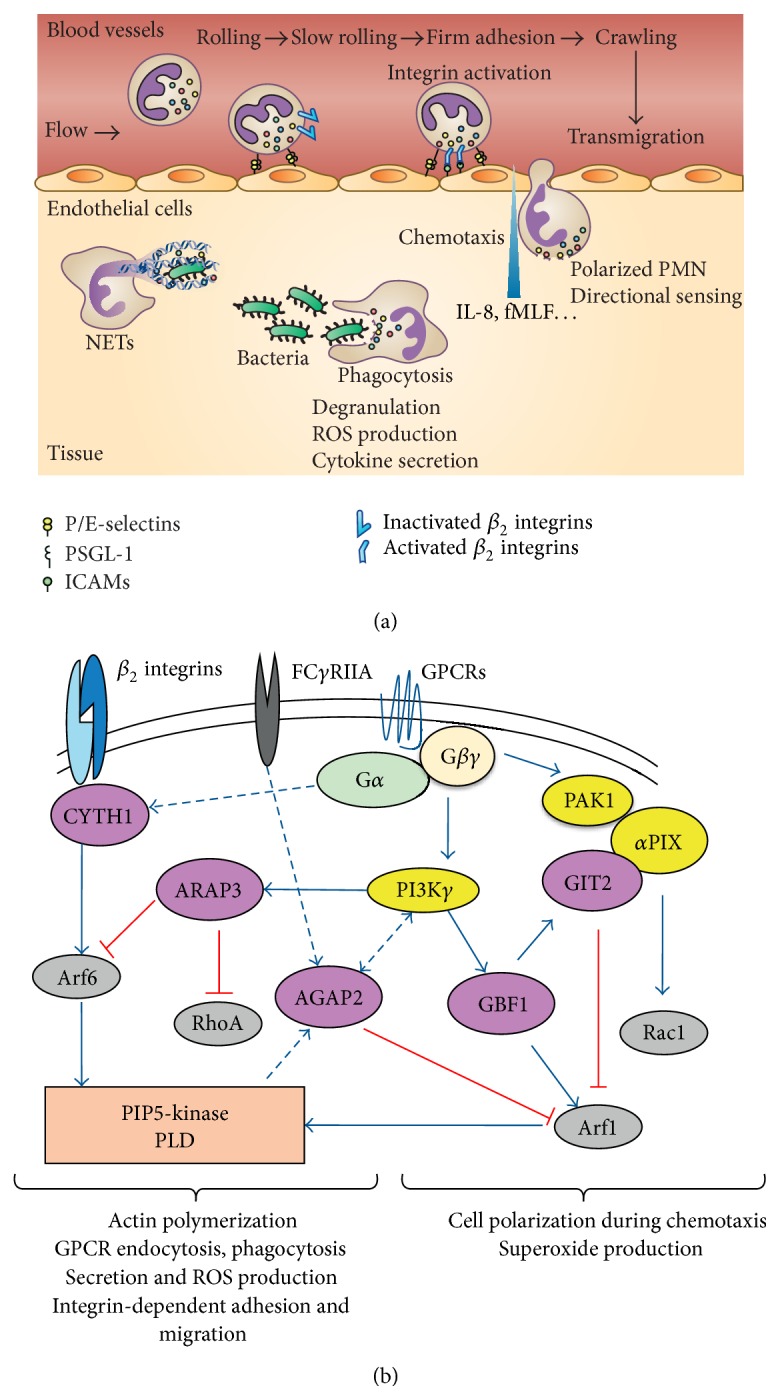
Main steps in PMN transmigration and regulation of PMN functional responses by Arfs and their regulators. (a) Schematic representation of PMN extravasation in infectious and noninfectious diseases. The first contact with endothelial cells is mediated by engagement of selectins with their counterreceptor P-selectin glycoprotein ligand-1 (PSGL-1) which results in capture and rolling of PMNs. Activation of PMNs by selectins and the different inflammatory signals like chemokines while rolling induces activation of the *β*_2_ integrins (LFA-1 and Mac-1) and slow rolling. Binding of activated *β*_2_ integrins to their counterreceptors ICAMs on endothelial cells induces PMN arrest due to firm adhesion and Mac-1-dependent crawling. Polarization of PMNs toward the chemoattractant source (i.e., cytoskeletal rearrangement, recruitment of regulators of Arfs, and activation of PI3K*γ*, Arf1, and NADPH oxidase at the leading edge) initiates directional sensing and transmigration across the vascular endothelium. PMNs are guided by the gradient of chemoattractant factors and after arriving at the site of infection or tissue injury, the cells initiate phagocytosis or NETosis to kill pathogens and remove cellular debris. PMN granules are schematically represented by colored circles. (b) Signalling pathways downstream of GPCRs, Fc*γ* receptor IIA (Fc*γ*RIIA), and *β*_2_ integrins by which Arfs and their regulators are thought to regulate PMN functional responses are presented schematically. Green arrows indicate direct activation either through lipid-protein or through protein-protein interactions, and negative feedback mechanisms are highlighted in red. Where direct interactions have not been established and/or the signalling mechanisms are unclear, lines are dotted. Cross talk between Arf and Rho family GTPases mediated by ARAP3 and the p21 protein- (Cdc42/Rac-) activated kinase 1 (PAK1)/PAK-interacting Exchange Factor alpha (*α*PIX) signalling complex is also shown.

Rapid recruitment of innate immunity cells such as polymorphonuclear neutrophils (PMNs) is a critical component of pathogen killing and removal during infection. PMNs are generated from hematopoietic stem cells located in the bone marrow. A normal adult is estimated to produce about 100 billion of these PMNs daily. As they differentiate, the cells begin to move toward the venous sinusoids prior to migration across the sinusoidal endothelium to reach the vascular lumen. These terminally differentiated cells have a short life [1] but nevertheless represent the most abundant leukocyte species in the circulation. PMNs are the first leukocytes to migrate from the blood to inflammatory sites [2, 3]. Following their activation by various proinflammatory cytokines such as IL-8, TNF*α*, or IL-1*β* secreted by resident macrophages, PMNs start rolling along the vessel wall, followed by firm arrest and transmigration through the inflammatory vascular endothelium [4–6]. Once in the extravascular environment, PMNs interact with extracellular matrix proteins and migrate along a chemotactic gradient to reach the site of injury [7]. At the site of infection, PMNs begin phagocytosis and killing of pathogens through production of toxic reactive oxygen species (ROS), secretion of lysosomal enzymes, and formation of NETs (Figure 1(a)) [2, 8]. Activated PMNs also regulate the innate and the adaptive immune responses by secretion of various cytokines and chemokines, such as IL-1, IL-6, IL-8, TNF*α* [2, 9–11], and lipid mediators as well [12]. The interaction of PMNs with their environment is an indispensable determinant that tailors their functional responses, including correct timing of events leading to activation. The mechanisms that contribute to the maintenance of PMN homeostasis under normal and inflammatory conditions are tightly regulated through integration of external signals picked up by their transmembrane receptors. These receptors mediate intracellular signalling cascades through activation of two superfamilies of G proteins, the heterotrimeric G proteins, and the RAS superfamily of small monomeric GTPases [13, 14]. Whereas heterotrimeric G proteins directly interact with and are activated following stimulation of so-called G protein-coupled receptors (GPCRs) such as formyl-peptide receptors or CXCR1 chemokine receptor, small GTPases are generally activated by other regulatory proteins downstream of many transmembrane receptors (Figure 1(b)).

The RAS superfamily comprises more than 150 members divided into six subfamilies: Ras, Rho, Ran, Rab, Rheb, and Arf [15]. Small GTPases are molecular switches that exist in an active “on” form when bound to Guanosine-Triphosphate (GTP) and an inactive “off” conformation when bound to Guanosine-Diphosphate (GDP) [15, 16]. The activation-deactivation cycle is coordinated by three different factors. The Guanine Nucleotide Exchange Factors (GEFs) catalyze the removal of GDP and allow GTP binding to the conserved guanine nucleotide binding site of small GTPases. The binding of GTP rigidifies small GTPases in an active conformation that interacts with specific effector proteins and engages a limited number of downstream effects. Small GTPases have low intrinsic GTPase activity and need help from GTPase Activating Proteins (GAPs) to hydrolyse GTP and return to the inactive GDP-bound conformation [17, 18]. Some GTPases of the Rho and Rab family are also regulated by Guanine Nucleotide Dissociation Inhibitors (GDIs) that remove small GTPases from the membranes and sequester them in the inactive state as a cytosolic heterodimer.

Small GTPases are key elements of downstream signalling pathways regulating multiple effector proteins and functional responses of cells [21]. Numerous studies, including those from our laboratory, have shown that Arf proteins activate phospholipase D (PLD) and phosphatidylinositol 4-phosphate 5-kinase (PIP5-kinase) and regulate various PMN functions such as superoxide production, degranulation, and chemotaxis (Figure 1(b) and Table 1). In this review, we will focus on the Arf GTPases and their regulators in relation to PMN functional responses.

## 2. Arfs

The Arf family was first identified and named according to its function as a cholera toxin cofactor stimulating the ADP-ribosylation of G protein G*α* subunit [22]. Arf GTPases are ubiquitously expressed and their sequences are highly conserved among eukaryotes. They are divided into three classes based on sequence homology. Class I includes Arf1, Arf2 (absent in humans), and Arf3, class II comprises Arf4 and Arf5, and Arf6 is the only representative of class III [23, 24]. We cannot overlook the fact that small G proteins sharing structural features with Arfs also include Arf-like (Arl) proteins, Ras-related protein 1 (Sar1), and Arf-related protein 1 (Arfrp1) [25]. Like all small GTPases, Arfs are on-off molecular switches regulated by specific GAPs and GEFs. In contrast to other small G proteins that undergo posttranslational modification (prenylation or isoprenylation) at their C-terminus, Arfs share N-terminal amphipathic helix with a myristoylated N-terminal glycine residue. GTP binding to Arfs induces conformational changes in switch 1 and switch 2 regions that bind effector proteins [26], as well as a reorientation of the amphipathic helix that favours interaction with the membrane and insertion of the lipid tail into the phospholipid bilayer [27]. Arf1 and Arf3 are 3–10-fold more highly expressed than other Arfs in cells [28]. Arfs also have different distribution in the cells, which is thought to stem from the individual protein environment. Although classes I and II Arfs are mainly localized to the ER-Golgi, Arf1 and Arf3 are released in the cytosol, whereas Arf4 and Arf5 can associate with the Golgi and the trans-Golgi network (TGN) in their GDP-bound state [29]. Studies have determined that the GDP-GTP cycle of Arf6 takes place at the plasma membrane and that some GTP-binding defective mutants of Arf6 are trapped in endosomes [30, 31]. The two that have been the most studied are Arf1 and Arf6 (Table 1).

### 2.1. Arf Class I

Arf1 was first reported to regulate intracellular vesicular traffic from the Golgi to the endoplasmic reticulum (ER) and between Golgi cisternae, through the recruitment of clathrin and nonclathrin coats to membrane, a first step in the budding of transport vesicles [50–52]. In addition to stimulating PLD, Golgi-associated Arfs recruit phosphatidylinositol 4-kinase and PIP5-kinase to maintain the structure and the dynamics of the Golgi apparatus through local synthesis of phosphatidylinositol-4-phosphate and phosphatidylinositol-4,5-bisphosphate (PtdIns(4,5)P_2_) [53]. Arf1 and Arf3 are required for the integrity of recycling endosomes but seem dispensable for the retrograde transport from endosomal compartments to the TGN [54]. Arf3 regulates trafficking of Toll-like receptor 9 (TLR9) [55].

In PMNs, Arf1 was shown to mediate formyl-methionyl-leucyl-phenylalanine (fMLF) dependent activation of PLD [56]. Studies from our laboratory have shown that the particulate agonist monosodium urate crystals [32], fMLF [33], and leukotriene B_4_ induce Arf1 recruitment to membranes and PLD activation in PMNs [34]. A study using cytosol-depleted HL-60 cells suggested that Arf1 and phosphatidylinositol transfer protein (PITP) restore secretory function in cytosol-depleted cells by promoting PtdIns(4,5)P_2_ synthesis [57]. PtdIns(4,5)P_2_ synthesis could also be dependent on Arf1-mediated activation of PIP5-kinase in HL-60 cells [36]. In PMNs, Arf1 was shown to bind to complement receptor type 1 (CR1) storage vesicles and was suggested to play a role in regulation of their transport [58]. Though activated Arf1 has been shown to recruit arfaptin-1 and arfaptin-2 to Golgi membranes and has been suggested to regulate Golgi function in HL-60 cells [35], more recent studies showed that Arl1 but not other Arf proteins determine the association of arfaptins with and the biogenesis of secretory granules at the trans-Golgi in cells [59, 60].

### 2.2. Arf Class II

Less is known about the function of Arf class II. Arf4 and Arf5 play roles in transport mechanisms between the endoplasmic reticulum Golgi intermediate compartment (ERGIC) and the Golgi [61]. Arf4 was reported to regulate transport of ciliary cargoes [62], and the CREB3-Arf4 signalling cascade was suggested to be part of a Golgi stress response to pathogens [63]. In HeLa cells, Arf5 was reported to regulate internalization of the *α*_5_ *β*_1_ integrin and clathrin-mediated endocytosis of specific cargoes [64]. Though quantitative proteomics studies have identified Arf4 and Arf5 in PMNs [47, 65], the functions of Arf class II in PMNs have not been investigated.

### 2.3. Arf Class III

Arf6, the Arf GTPase most distantly related to Arf1, has been localized to the plasma membrane and endosomal compartments [31]. This GTPase has been implicated in different signalling pathways and in a wide diversity of cellular functions such as actin cytoskeleton remodelling, phagocytosis, endocytosis, membrane receptor recycling, and intracellular transport [52, 66–70]. Overexpression of Arf6 and of its regulators in metastatic cancers suggests important roles in regulating adhesion, migration, and invasive behaviour of cancer cells [71–73]. For example, in epithelial cells, E-cadherin is targeted by Arf6 to adherens junctions for maintaining barrier permeability, epithelial cell morphology, and polarity [74, 75]. In immune cells such as macrophages, a spatiotemporal recruitment of Arf6 to phagosomes regulates Fc*γ* receptor-dependent phagocytosis [76–79]. This GTPase is also involved in endocytosis and recycling of various GPCRs [80–82] such as mu-opioid [83] and *β*2-adrenergic [84] receptors, or growth factor receptors as well [85]. Moreover, it is important to mention that Arf6 plays a major role in the signalling pathway of Toll-like receptor 4 (TLR4) and TLR9 [86, 87].

The expression of Arf6 in PMNs and neutrophil-like cells such as differentiated HL-60 or PLB-985 myeloid leukemia cells has been previously reported [36–38]. Arf6 protein is four to five times more abundant in these myeloid leukemia cells when compared to human PMNs [37]. In PMNs or differentiated PLB-985 cells, Arf6 plays roles in the signalling pathways elicited by the chemotactic peptide fMLF [37, 38]. PLB-985 cells, overexpressing the Arf6 (Q67L) mutant defective in GTP hydrolysis or the Arf6 (T27N) mutant defective in GTP binding, show increased and decreased NADPH oxidase activity, respectively [38]. Silencing of Arf6 in PLB-985 cells also reduces fMLF-mediated production of superoxide and PLD activation as well [37]. Inhibition of superoxide production by Arf6 mutants could be due to reduced PLD activity since overexpression of the Arf6 (N48R) mutant defective in PLD activation also reduced fMLF-induced NADPH oxidase activity in PLB-985 cells [38]. In addition to PLD, PIP5-kinase was reported to be a downstream effector of Arf6 [88, 89]. In this context, it is important to highlight that Arf proteins can be depleted from HL-60 cells by permeabilization and that addition of Arf6 to permeabilized cells contributes to the regulation of PdtIns(4,5)P_2_ synthesis at the plasma membrane by directly activating PIP5-kinase [36]. Altogether, these studies suggest that remodelling of membrane phospholipids by Arf6-mediated activation of PLD enzymes and PIP5-kinase would regulate PMN functional responses such as NADPH oxidase activity, phagocytosis, and degranulation [90–92]. Direct demonstration of a role for Arf6 in PMN phagocytosis and degranulation awaits characterization of Arf6 knockout PMNs.

## 3. Arf GEFs

GEFs can signal from plasma membrane receptors directly to small GTPases, and in some cases GEFs serve as GTPase effectors or adaptor proteins that facilitate activation of other small GTPase family members [93]. The human genome contains GEFs that are family specific but some GEFs are highly specific toward one GTPase. Mammalian Arf GEFs comprise a family of 16 proteins [25]. The first characterized Arf GEFs comprise the yeast Gea1p and in mammals cytohesin-2 and BIG1 [94–96]. Although Arf GEFs show different substrate specificities, they share a conserved 200-amino acid region called the Sec7 domain that catalyzes the exchange of nucleotides. Some but not all Sec7 domains are the target of the drug Brefeldin A (BFA) [97]. Arf GEFs contain other motifs, like the Pleckstrin Homology (PH) domain involved in protein targeting to membranes through binding to polyphosphoinositides, homology downstream of Sec7 (HDS) lipid-binding domains, or SH2 and SH3 domains related to protein-protein interactions [25]. Arf GEFs are classified into six evolutionarily conserved families as follows: GBF1, BIG, PSD, IQSEC, cytohesins, and FBXO8.

The Golgi-specific BFA resistance factor 1 (GBF1) and the two yeast GBF1 orthologs, Gea1 and Gea2, localize in the Golgi. Lipid binding to the HDS1 domain immediately downstream of the Sec7 domain is sufficient for targeting GBF1 to lipid droplets and Golgi membranes [98]. GBF1 recruits the COPI coat to the cis-Golgi [99–101].* In vitro*, GBF1 acts preferentially on Arf5 [102]. In differentiated HL-60 cells, GBF1 has been reported to activate Arf1 in response to stimulation with fMLF [44]. Upon stimulation, Arf1 and GBF1 are relocalized from the Golgi to the leading edge of migrating cells in a phosphatidylinositol 3-kinase- (PI3K-) dependent manner. The silencing of GBF1 in HL-60 cells abrogates cell polarisation, direction sensing, and superoxide production induced by fMLF [44].

The BIG (BFA inhibited GEF) family of Arf GEFs comprises BIG1 and BIG2 in mammals. BIG2 has been shown to activate class I Arfs (Arf1 and Arf3) and to localize to the TGN and recycling endosomes [103–106]. BIG1 and BIG2 have redundant functions and are important determinants of Arf-based membrane traffic between the TGN and late endosomes [106]. In addition to lipid binding, the HDS1 domain of Sec7, the yeast orthologue of BIG2/BIG2, is important to localize this Arf GEF to Golgi membrane compartments on which Arf1 has already been activated [107]. A cascade in which GBF1-activated Arf4 and Arf5 regulate the recruitment of BIG1 and BIG2 to the TGN has been reported [108]. This study provides a mechanistic basis for the effects of a combination of Arf class I and class II knockdowns on Golgi morphology [61]. Although BFA has been reported to inhibit fMLF-mediated production of superoxide in PMNs, the BFA sensitive Arf GEF involved in this effect, if any, has not been characterized [109].

The PSD family of Arf GEFs, also known as EFA6 (Exchange Factor for Arf6), comprises four paralogs in vertebrates (EFA6A, EFA6B, EFA6C, and EFA6D) [110]. EFA6 is plasma membrane-targeted through interaction with PtdIns(4,5)P_2_ and F-actin [111, 112]. EFA6 is involved in cytoskeletal rearrangement and clathrin-mediated endocytosis [112, 113]. There is no report on expression of EFA6 by PMNs.

The BFA resistant Arf GEFs (BRAGs) or IQSEC family contains three members in vertebrates [25]. BRAG1/2 were found associated to endosomes and were found to localize with Arf6 at the cell periphery [114–116]. In addition to Arf6, BRAG2 was reported to activate Arf4 and Arf5 and to regulate Arf5-dependent internalization of *β*_1_ integrins in the clathrin-coated pits [64]. Other studies have shown that BRAG2 plays a role in cell adhesion and phagocytosis through regulation of *β* integrin trafficking in epithelial cells and monocytes, respectively [117, 118]. There is no information on BRAG proteins in PMNs.

Other Arf family GEFs include Sec12, a type II ER membrane protein that is a specific Sar1 GEF [119], and the F-box protein 8 gene family (FBXO8) [120]. FBXO8 might not function as a GEF but as a factor that controls the intracellular levels of Arf6 protein through ubiquitinylation-mediated proteasomal degradation [121].

The cytohesin (CYTH) family is represented by four BFA-insensitive Arf GEFs in vertebrates. The structural organization of CYTHs includes N-terminal coiled-coil domain involved in CYTH dimerization or protein-protein interaction, the Sec7 domain, and a C-terminal PH domain [122, 123]. The affinity of their PH domain for PtdIns(4,5)P_2_ and phosphatidylinositol 3,4,5-trisphosphate (PtdIns(3,4,5)P_3_) is an important determinant for CYTH localization and/or recruitment to the plasma membrane [123]. Studies from our laboratory have reported the expression of cytohesin-2/ARNO (CYTH-2) and cytohesin-3 (CYTH-3) in undifferentiated HL-60 or PLB-985 cells and a strong induction of cytohesin-1 (CYTH-1) expression during granulocytic differentiation [20, 39]. In differentiated HL-60 cells, stimulation with fMLF induced a PI3K-independent and PI3K-dependent membrane recruitment of CYTH-1 and CYTH-2, respectively [20]. PMNs express mainly CYTH-1. We have previously shown that pharmacological inhibition of CYTH-1 with SecinH3 inhibited fMLF-mediated membrane translocation of Arf6 and Arf1 and activation of Arf6 in PMNs [37]. Studies using SecinH3 in PMNs and PLB-985 cells overexpressing CYTH-1 or silenced for Arf6 have highlighted a role for the CYTH-1-Arf6 signalling axis in PLD activation and two major bactericidal functions, degranulation and NADPH oxidase activity [37]. Furthermore, pharmacological inhibition or silencing of CYTH-1 was shown to reduce the internalization of FPRL-1 (formyl-peptide-like receptor 1) in fMLF activated granulocytes [39].

CYTH-1 was initially characterized as a positive regulator of the *β*_2_ integrin LFA-1 (lymphocyte function antigen-1) functions in lymphocytes [40–42]. In our laboratory, we showed that overexpression of CYTH-1 in PLB-985 cells increases LFA-1-dependent adhesion to endothelial cells [43]. In contrast, PLB-985 cells silenced for CYTH-1 and PMNs treated with SecinH3 show decreased LFA-1-dependent adhesion to endothelial cells [43]. Further studies from our laboratory also documented that CYTH-1 associates with and restrains the activation of the *β*_2_ integrin Mac-1 (macrophage antigen-1), thereby having a negative impact on PMN adhesion to fibrinogen, chemotaxis, and phagocytosis [39]. Altogether, these studies suggest that CYTH-1 in PMNs differentially regulates the activation of the *β*_2_ integrins LFA-1 and Mac-1 [42].

## 4. Arf GAPs

GTPase Activating Proteins (GAPs) are proteins that accelerate the intrinsic GTP hydrolysis activity of small G proteins. The Arf GAPs contain a characteristic domain of about 130 amino acids, which has been shown to be the minimum unit with GAP activity [124]. The GAP domain has a zinc-finger structure that is unique to Arf GAPs, but similar to other GAPs for the Ras and Rho families of small GTPases, there is a conserved arginine that is essential for the catalytic activity of the so-called “arginine finger” [125, 126]. The human genome is predicted to encode thirty-one proteins containing the Arf GAP domain [127]. Mammalian Arf GAPs are selective for one or more Arfs [128] but are not active on Sar1 or Arl proteins. However, GAP selectivity* in vivo* is likely to depend on the localization of Arf GAPs and Arfs in cells, as well as on composition and shape of lipid bilayer membranes. In addition to the GAP domain, these proteins have a variety of other domains involved in intramolecular, protein-protein, and protein-lipid interactions [129]. The Arf GAPs have been classified into two major groups according to the domain structure [130]. The ArfGAP1-type with N-terminal GAP domain includes the ArfGAP, SMAP, ADAP, and GIT protein subtypes. The AZAP-type with a GAP domain in a sandwich between a PH domain and the ankyrin (ANK) repeat motif comprises ASAP, ACAP, AGAP, and ARAP subtypes.

ArfGAP1 was the first Arf GAP identified in mammals [124]. Its GAP activity is stimulated by diacylglycerol [131]. ArfGAP1 contains two motifs termed amphipathic lipid packing sensor (ALPS) that allow binding to liposomes [132]. The ALPS motifs for Golgi localization make ArfGAP1 activity extremely sensitive to membrane lipid curvature [132, 133]. The primary function attributed to ArfGAP1 is regulation of COPI vesicle biogenesis by stimulating the hydrolysis of GTP bound to Golgi Arfs [124, 134]. ArfGAP2 and ArfGAP3 lack the ALPS motif of ArfGAP1 but instead possess dilysine retrieval motifs that confer Golgi localization through direct interaction with the COPI coat [135–137]. ArfGAP2 and ArfGAP3 are key components of the COPI coat lattice and coatomer-induced GAP activity may be required for proper vesicle formation [135–137]. There are no reports on ArfGAP1/2/3 in PMNs.

The SMAP subfamily comprises two members, SMAP1 and SMAP2 [138, 139]. SMAP protein structure includes a clathrin box that binds clathrin heavy chains and regulates the trafficking of clathrin-coated vesicles [138, 139]. SMAP1 was reported to be an Arf6 GAP regulating Arf6-dependent endocytosis of transferrin and E-cadherin receptors [140], whereas SMAP2 was involved in Arf1-dependent membrane trafficking between early endosomes and the TGN [141]. Although SMAP1 and SMAP2 were initially shown to have distinct functions, the proteins were also reported to interact which each other and to regulate transferrin receptor endocytosis [142]. SMAP1 deficient mice are more prone to develop myelodysplasia [143]. There are no reports, as of yet, on the expression of SMAP proteins in human PMNs and in human tumor-derived myeloid cell lines.

**Figure 2:**
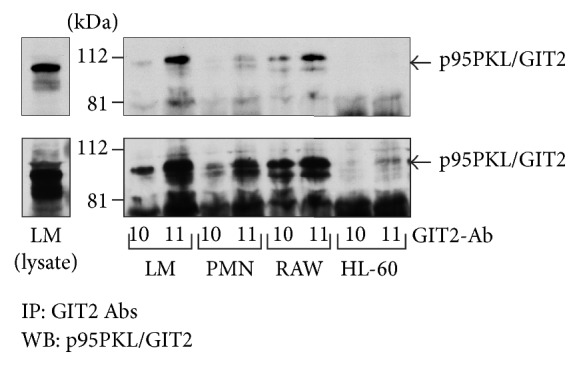
Expression of GIT2 in immune cells. RAW264 macrophages (1.5 × 10^7^ cells), human PMNs (3 × 10^7^), human lymphocytes/monocytes (LM, 3 × 10^7^), and dimethyl sulfoxide-differentiated HL-60 cells (3 × 10^7^) were mixed with an equal volume of boiling denaturing buffer and cell lysates were processed essentially as described by Marcil et al. [19]. The supernatants were then filtered through Sephadex G-10 columns to remove the denaturing agents and 0.1% Nonidet P-40, 20 *μ*g/mL aprotinin, 20 *μ*g/mL leupeptin, and 5 *μ*L of bovine serum albumin (0.01% w/v) were added to the eluates. Samples were precleared with protein A-Sepharose and subsequently used for overnight immunoprecipitation with the polyclonal GIT2 antibodies 10 and 11 (5 *μ*L). The beads were washed three times with ice-cold nondenaturing lysis buffer containing 1% Nonidet P-40 and boiled for 7 min at 100°C in 2x Laemmli's sample buffer as described previously [19]. Immunoprecipitated proteins were electrophoresed on 10% SDS-PAGE and proteins were transferred to Immobilon PVDF membrane (Millipore Corp., Bedford, MA, USA). Membranes were incubated with the p95PKL/GIT2 antibody (P94020; 1 : 1500) from Bection Dickinson (Mississauga, ON, Canada) and exposed to peroxidase-conjugated anti-rabbit IgG (1 : 20,000) for 1 h at 37°C. The membranes were covered with ECL+ detection reagents. Images were obtained by exposing Kodak X-Omat film to membranes for 20 sec (upper panel) and 5 min (lower panel).

The GIT subfamily includes the two structurally related proteins GIT1 and GIT2 [127]. They possess the zinc-finger motif required for their GAP activity on Arf6 [144]. Though GIT proteins have no PH domain, their GAP activity was reported to be stimulated by PtdIns(3,4,5)P_3_ [144]. GIT1 interacts with various GPCRs to regulate their endocytosis via the clathrin pathway in a G protein-coupled receptor kinase, *β*-arrestin, and dynamin-dependent manner [145, 146]. It is worth highlighting that GIT proteins can form complexes with PIX, a GEF specific for Rho GTPases Rac2 and Cdc42 [147]. GIT/PIX complexes regulate Cdc42/Rac-dependent activation of p21-activated kinase 1 (PAK1), a protein involved in microtubule-mediated focal adhesion disassembly [148]. GIT2 and a splice variant named GIT2-short have been characterized, with the expression of the latter being restricted to immune cells [149]. Overexpression of GIT2-short was reported to cause redistribution of Golgi protein *β*-COP, to affect the subcellular localization of paxillin, and to reduce the levels of actin-based fibers [150]. GIT2 is expressed in human lymphocytes and/or monocytes, mature PMNs, HL-60 promyelocytic leukemia cells, and the rat macrophage cell line RAW264 (Figure 2). In PMNs obtained from GIT2-deficient mice, Arf1 was reported to be hyperactivated in response to stimulation with fMLF [45]. GIT2 deficiency was associated with reduced directional migration to fMLF and enhanced production of superoxide even if NADPH oxidase polarization at the leading edge of migrating PMNs was lost [45]. Interestingly, the Arf GEF GBF1 has been suggested to control the activation of Arf1 and to target p22phox and GIT2 to the leading edge of chemotaxing PMNs [44].

The ASAP subtype includes ASAP1, ASAP2, and ASAP3 [127]. This family possesses BAR, PH, and Arf GAP domains in tandem. More information on the domain structure of ASAPs can be found elsewhere [25, 127]. ASAP1 and ASAP2 have PIP_2_-dependent GAP activity and both act on Arf1 and Arf5 and only weakly on Arf6* in vitro* [151]. ASAP1 was involved in the regulation of cytoskeletal remodeling [152]. It was shown that ASAP1 is in an autoinhibited conformation in its native state. This is possibly due to intramolecular interaction between the BAR and PH domains, which affects GAP activity independently of the property of BAR domain in mediating association of ASAP1 with membranes [153]. Although ASAP1 and ASAP2 were detected in PMNs using proteomic analyses [46], their subcellular distribution and biological functions remain open questions.

**Figure 3:**
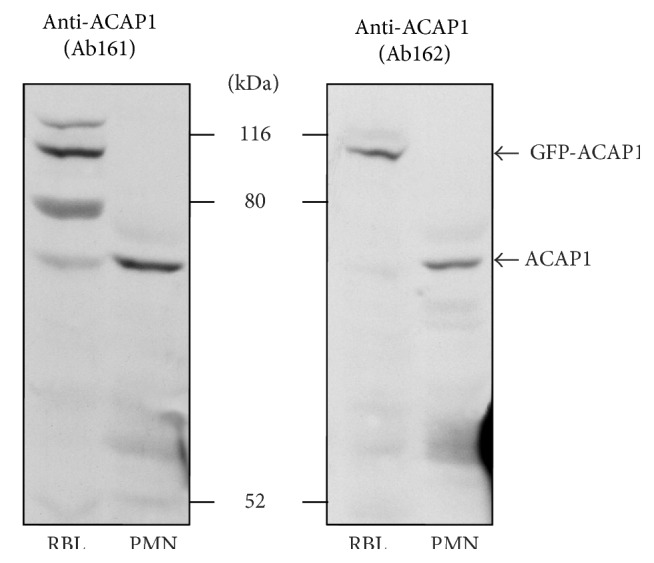
Expression of ACAP1 by human PMNs. Lysates from RBL-2H3 cells (0.5 × 10^6^) overexpressing ACAP1-GFP and human PMNs (2 × 10^6^) were subjected to 8% SDS-PAGE and proteins were transferred to Immobilon PVDF membrane. Membranes were incubated with our homemade polyclonal antibodies (serums 161 and 162) against ACAP1 (1 : 1000) and exposed to peroxidase-conjugated anti-rabbit IgG (1 : 20,000) for 1 h at 37°C. The membranes were covered with ECL+ detection reagents. Images were obtained by exposing Kodak X-Omat film to membranes.

The ACAP family comprises three members, with ACAP1 and ACAP2 being the best characterized. ACAP1 and ACAP2 are activated by PtdIns(4,5)P_2_ and PtdIns(3,5)P_2_ [154]. ACAP homologs in* Dictyostelium* were shown to affect the actin cytoskeleton and to regulate cytokinesis [155, 156]. However, their role in chemotaxis is still unclear [154–156]. A recent study suggested a role for ACAP2 in Fc*γ*R-dependent phagocytosis in macrophages [157]. Proteomic analysis has detected ACAP1 in PMNs [46]. Polyclonal ACAP1 antibodies generated in our laboratory detected a protein of about 75 kDa in PMNs and ACAP1-GFP overexpressed in RBL-2H3 cells (Figure 3). But to our knowledge, no one has yet explored the function of this Arf GAP in PMNs.

The three members of the ARAP subtype have Rho GAP domain in addition to an Arf GAP domain with multiple PH domains that recognize PtdIns(3,4,5)P_3_ [25, 127, 158]. ARAP1 regulates endocytosis of epidermal growth factor receptor (EGFR) [159, 160]. Receptor internalization requires the interaction of ARAP1 with multiple proteins such as CIN85 and its phosphorylation by Src kinase [160, 161]. Further investigation is required to assess the impact of phosphorylation and protein-protein interaction on ARAP1 GAP activities. ARAP1 also regulated the filamentous-actin ring structure size of circular dorsal ruffles in NIH 3T3 cells through an Arf1/5-dependent mechanism [162]. ARAP2 was shown to regulate focal adhesion dynamics using Arf6 [163].* In vitro* and* in vivo* ARAP3 has been reported to be a specific Arf6 GAP [158, 164]. ARAP1 and ARAP3 were detected in neutrophils using proteomics methods [46, 47]. Recent studies using an inducible ARAP3 KO mouse model suggest that this GAP affects *β*_2_ integrin functions and several biological responses dependent on integrin activation such as adhesion-dependent ROS formation, granule release, and chemotaxis through modulation of RhoA but not of Arf6 activation [48, 49].

**Figure 4:**
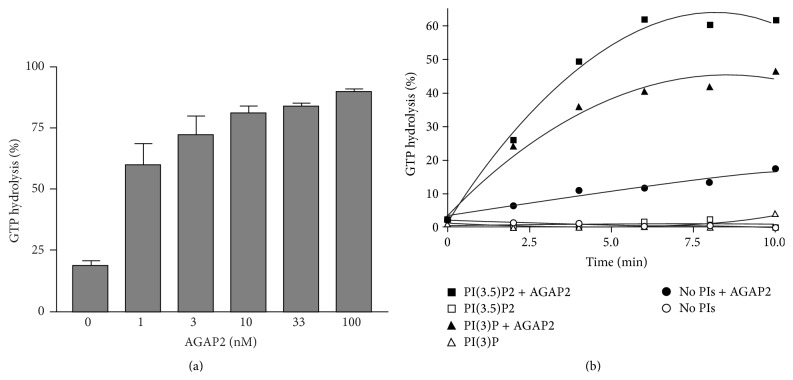
AGAP2 efficiently stimulates GTP hydrolysis on Arf1 and GAP activity is stimulated by products of PI3K, PtdIns(3)P, and PtdIns(3,5)P_2_. Recombinant myristoylated Arf1 was purified from* E. coli* as previously described [20]. AGAP2 cDNA was inserted into the pACHLT-A baculovirus shuttle vector and cotransfected with linearized BaculoGold viral DNA into sf9 cells. Culture supernatants were used to infect sf9 cells with an MOI of 10. Insect cells were collected 48 h after infection and His6-AGAP2 was purified from sf9 lysates by chromatography on Ni-trap columns. (a) GTP*α* ^32^P was loaded onto Arf1 in the presence of 1 mg/mL of liposomes composed of phosphatidylcholine, phosphatidylethanolamine, and phosphatidylserine (molar ratio 40.55 : 31 : 28.45) for 30 min at 30°C. AGAP2 at the indicated concentrations was mixed with 0.3 *μ*M GTP*α* ^32^P-loaded Arf1 and incubated for 30 min at 30°C in GAP buffer (20 mM Tris pH 8.0, 2 mM DTT, 100 mM NaCl, 1 mM MgCl_2_, and 100 *μ*g/mL liposomes). (b) GTP*α* ^32^P was loaded onto Arf1 in the presence of 1 mg/mL of liposomes composed of phosphatidylcholine, phosphatidylethanolamine, phosphatidylserine (molar ratio 40.55 : 31 : 28.45), and liposome-supplemented PtdIns(3)P or PtdIns(3,5)P_2_ (molar ratio 37.4 : 28.5 : 26.2 : 7.9) for 30 min at 30°C. AGAP2 (10 nM) was mixed with 0.3 *μ*M GTP*α* ^32^P-loaded Arf1 and incubated at 30°C in GAP buffer for indicated time points. Reactions were stopped by dilution in ice-cold stop buffer (20 mM Tris pH 8.0, 1 mM DTT, and 10 mM MgCl_2_). Samples were filtered on Gelman GN-6 membranes and bound nucleotides were eluted with 2 M LiCl. GTP was separated from GDP by chromatography using polyethylenimine cellulose TLC plates developed in 1 M LiCl/1 M formic acid. The GTP*α* ^32^P/GDP*α* ^32^P ratios were calculated after exposure of TLC plates to a phosphorimager.

**Figure 5:**
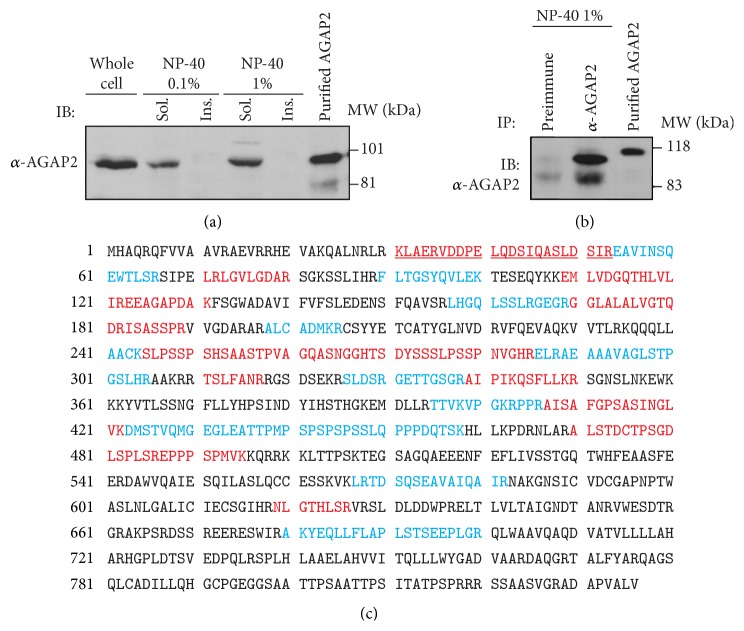
Expression of AGAP2 protein in PMNs. (a) Cells were lysed in nondenaturing isotonic (1% NP-40) or hypotonic (0.1% NP-40) lysis buffer. (b) PMNs were lysed in nondenaturing isotonic lysis buffer and AGAP2 was immunoprecipitated with preimmune serum or immune serum raised against AGAP2. Cell lysates derived from 1.5 × 10^6^ PMNs (a) and immunoprecipitates (b) were resolved by SDS-PAGE. Purified His6-tagged AGAP2 was used as a control. (c) Amino acid sequence of human AGAP2 (NCBI Reference Sequence: NP_055585.1). AGAP2 was immunoprecipitated from PMNs (4 × 10^7^/mL) as described in (b). The samples were resolved using 7.5–20% SDS-PAGE and the gel was stained with SYPRO Ruby. Bands of interest were analysed by mass spectrometry. The peptides identified by mass spectrometry are in red or blue. Peptides unique to AGAP2 are underlined.

In humans, 11 genes are predicted to encode for AGAP-type Arf GAPs [127], with AGAP1 and AGAP2 being the most studied. AGAP1/2 have high GAP activity toward Arf1 and Arf5 and weak activity towards Arf6 [165, 166]. GAP activity is stimulated by PtdIns(4,5)P_2_ and phosphatidic acid as well [165, 166]. The AGAP2 gene encodes for three protein isoforms; PIKE-L and PIKE-S, which are restricted to brain, whereas PIKE-A (AGAP2) is more ubiquitously expressed [167, 168]. As shown in Figure 4(a), purified recombinant AGAP2 is a very potent Arf1 GAP. GAP activity is strongly stimulated by PtdIns(3)P and PtdIns(3,5)P_2_, the products of PI3Ks (Figure 4(b)). AGAP2 was reported to colocalize with AP-1 and transferrin receptors on recycling endosomes, and, together with Arf1, to regulate retrograde trafficking between early endosomes and the TGN [166, 169]. Moreover, AGAP2 plays a role in the signalling pathways and regulates the recycling of *β*_2_-adrenergic receptors [170]. During cell migration, AGAP2 was shown to promote focal adhesion disassembly through binding to and stimulation of focal adhesion kinase [171]. We generated polyclonal AGAP2 antibodies that detect a protein of about 90 kDa in PMNs (Figure 5(a)). The 90 kDa protein recovered in 1% NP-40 PMN lysates was immunoprecipitated by the AGAP2 antibody but not by the preimmune serum (Figure 5(b)). The band was analysed by mass spectrometry. Overall, 32 peptides covering 44% of the AGAP2 amino acid sequence were identified. Among these peptides, two were unique to AGAP2 and there were no signature peptides for AGAP1 or PIKE-L (Figure 5(c)). Taken together, the data indicate that AGAP2, but not AGAP1 or PIKE-L, was expressed in PMNs. This work is still in progress, but preliminary observations suggest that AGAP2 regulates phagocytosis independently of its GAP activity.

The ADAP subfamily includes two structurally related proteins with an Arf GAP and two PH domains in tandem [127]. ADAP1 is a brain specific PtdIns(3,4,5)P_3_-binding protein that functions as an Arf6 GAP* in vivo* [172, 173]. ADAP1 also serves as a scaffold in several signalling pathways through interaction with proteins such as F-actin, the kinesin family protein K1F13B, Ran binding protein in microtubule organizing center, *α*-tubulin, and PKC family members to name a few (reviewed in [174]). Through interaction with components of the cytoskeleton, ADAP1 has been suggested to regulate neuronal actin and vesicle transport along microtubules [173, 175]. ADAP1 has been shown to be involved in dendritic cell differentiation and development [176]. ADAP2 is a GAP selective for Arf6 that regulates cortical actin formation at the plasma membrane [177]. ADAP2 is abundantly expressed in fat, heart, and skeletal muscles [178] and was suggested to play a role in heart development [179]. A recent proteomic analysis of PMN subcellular fractions has identified ADAP2 in cell membranes [47].

## 5. Concluding Remarks

The presence of Arf proteins including Arf1, Arf3, Arf5, and Arf6 has been reported in PMNs and/or neutrophil-like cells. Arf1 and Arf6 regulate various biological responses through stimulation of the lipid remodelling enzymes PLD and PIP5-kinase (Table 1). Pharmacological approaches and the use of neutrophil-like cells have permitted the investigation of the role of Arf6 in PMN functional responses such as NADPH oxidase activity, phagocytosis, and degranulation. Several regulators of Arfs have already been characterized. CYTH-1 and GBF1 are amongst the first Arf GEFs identified in PMNs. CYTH-1 was involved in PLD and NADPH oxidase activation, degranulation, and regulation of PMN adhesion through the *β*_2_ integrins Mac-1 and LFA-1. GBF1 is part of a signalling pathway coordinating cell polarisation, direction sensing, and superoxide production in response to stimulation with chemoattractants. There is still fragmentary information available on the biological functions of the various Arf GAPs expressed by PMNs. The best characterized include GIT2, a negative regulator of Arf1, and ARAP3, a dual Arf and Rho GAP. Whereas GIT2 is involved in PMN direction sensing and superoxide production, ARAP3 modulates *β*_2_ integrin functions and adhesion-dependent formation of ROS, granule content release, and chemotaxis (Table 1). Further studies on the Arf GAPs recently identified in human PMNs (ASAP1, ASAP2, ACAP1, ACAP2, ARAP1, ADAP2, and AGAP2) are required to understand the significance of these proteins in PMN biology.

## Figures and Tables

**Table 1:** Neutrophil functions modulated by Arfs and their regulators.

Detected in PMNs	Roles in PMNs (or PMN-like cells)	References
Arf1	PLD activation Secretion Golgi function	[32–35]

Arf6	PLD activation NADPH oxidase activity PIP5-kinase stimulation	[36–38]

CYTH-1	Arf6 activation PLD activation FPRL-1 internalization Regulation of *β*_2_ integrins Adhesion Chemotaxis Phagocytosis NADPH oxidase activity	[37, 39–43]

CYTH-2/3	Unknown	[20, 39]

GBF1	Activation of Arf1 Cell polarisation Direction sensing Superoxide production	[44]

GIT2	NADPH oxidase activity Directional migration Arf1 inactivation	[44, 45]

ASAP1/2	Unknown	[46]

ACAP1	Unknown	[46]

ARAP1	Unknown	[46, 47]

ARAP3	Regulation of *β*_2_ integrins Adhesion Chemotaxis ROS production	[46–49]

AGAP2	Phagocytosis	Unpublished

ADAP2	Unknown	[47]
